# An Immersive Virtual Reality Room to Enhance Positive Affect and Engagement in Nursing Home Residents with Neurocognitive and Psychological Disorders: A Feasibility Study

**DOI:** 10.3390/healthcare14050588

**Published:** 2026-02-26

**Authors:** Malgorzata Klass, Frédérick Dandler, Yaëlle Ducommun, Michel Hanset, Laurence Ruscart, Jean-Christophe Bier, Sandra De Breucker, Jennifer Foucart

**Affiliations:** 1 Research Unit in Cardio-Respiratory Physiology, Exercise & Nutrition, Faculty of Human Movement Sciences, Université Libre de Bruxelles, 1070 Brussels, Belgium; 2 Faculty of Human Movement Sciences, Université Libre de Bruxelles, 1070 Brussels, Belgium; frederick.dandler@ulb.be (F.D.); yaelle.ducommun@gmail.com (Y.D.); 3 Residential Care and Nursing Home La Cambre, 1170 Brussels, Belgium; michel.hanset@tklc.be (M.H.); laurenceruscart@gmail.com (L.R.); 4 Neurology Department, H.U.B.-Erasme Hospital, Université Libre de Bruxelles, 1070 Brussels, Belgium; jean-christophe.bier@hubruxelles.be; 5 Geriatric Department, Hôpitaux Universitaires de Genève (HUG), Rue Gabrielle Perret-Gentil 4, 1205 Geneva, Switzerland; sandra.debreucker@hug.ch; 6 Faculty of Medicine, Université Libre de Bruxelles, 1070 Brussels, Belgium; 7 Health and Movement Psychology Research Unit, Faculty of Human Movement Sciences, Université Libre de Bruxelles, 1070 Brussels, Belgium; jennifer.foucart@ulb.be

**Keywords:** virtual reality, dementia, CAVE, depression, anxiety, relaxation, social interaction, reminiscence

## Abstract

**Highlights:**

**What are the main findings?**

- Immersive VR room sessions were well tolerated, improved short-term positive affect, and fostered shared experiences and active engagement.
- Participants’ feedback highlighted the enjoyable, relaxing VR experience, noting the realism and aesthetics of the environments, which allowed them to travel virtually, reminisce about personal memories and experiences, and share these with the accompanying person.

**What are the implications of the main findings**

- The improvements introduced by the VR room align with recommendations for the development of immersive technologies for nursing home residents by providing realistic, nature-inspired environments, enhancing ease of use and interactivity, and enabling a multi-user experience that supports social interaction.
- By offering an emotionally positive and engaging experience, opportunities for meaningful social interaction, and greater freedom of movement than VR headsets, the VR room could represent an innovative tool for improving mood and supporting the development of novel cognitive interventions. However, further research is required to substantiate these potential benefits.

**Abstract:**

**Background/Objectives**: Older adults with neurocognitive and psychological disorders are often institutionalized in nursing homes, which negatively affects well-being and mood, and may accelerate cognitive decline. Immersive virtual reality (VR) is a promising non-pharmacological countermeasure, but VR-headset discomfort limits its usability in this population. Therefore, this study examined the tolerability and feasibility of an immersive VR room, which provides customizable interactive environments projected across four walls at 360° and enables shared experiences, to enhance positive affect and engagement in nursing home residents. **Methods**: Twenty nursing home residents were initially enrolled, and nineteen completed five 10 min sessions in the immersive VR room accompanied by a caregiver. State positive and negative effects were assessed using the visual analogue scale (VAS) and the Observed Emotion Rating Scale (OERS), and participants’ verbal feedback was collected during and after the sessions. **Results**: VAS scores indicated that VR room immersion was feasible and well-tolerated, with most participants feeling secure and experiencing increased positive affect during and just after the sessions. OERS scores and observations revealed frequent expressions of pleasure, interest, and active engagement with both the VR environments and the caregiver. Participants’ reports valued the enjoyable and relaxing experience provided by immersion in the VR room, noting the realism and aesthetics of the environments and nature-related elements, which allowed them to travel virtually and evoke personal memories. Conclusions: Immersive VR room sessions were well tolerated, enhanced positive affect, and may support cognitive functioning by fostering active engagement and social interaction. Given that this is a feasibility study with a small cohort and short follow-up, the present findings should be considered preliminary and confirmed in larger, controlled, longer-term studies.

## 1. Introduction

Demographic aging is one of the main challenges of this century. By 2050, the population aged over 60 is indeed expected to double, and the number of people aged 80 years or older is expected to triple [1]. Ageing is accompanied by an increased risk of developing depressive disorders and mild neurocognitive disorders (NCD) or major NCD (i.e., dementia). The average prevalence of late life depression in the community ranges between 8.4% and 16.6%, depending on age and sex, and rises to approximately 30% in nursing home residents [2,3]. Prevalence of dementia ranges from 7 to 9% in Europe for those aged 65 and over [4]. NCDs are characterized by a cognitive decline associated with behavioral and psychological symptoms (BPS), with the most frequently reported being apathy, dysphoria, aberrant motor behaviors, anxiety and depression [5,6,7]. Psychological symptoms in patients with mild NCD increase functional deficits and are a significant risk factor for progression to major NCD [8]. Older individuals with NCD and BPS are frequently institutionalized in long-term care institutions. Due to the stress induced by the relocation, loneliness, limited meaningful social interactions, and the monotony of everyday living, institutionalization is often accompanied by increased anxiety, depression, and accelerates cognitive decline [9,10,11].

BPSs are typically managed with pharmacological interventions, which unfortunately provide only modest relief, are associated with side effects, and may alter cognitive functioning [12]. Therefore, the use of non-pharmacological interventions (e.g., psychological, cognitive, exercise, sensory stimulation, etc.) as alternative approaches to pharmacological treatments is recommended to limit the side effects of psychotropic drugs and has been shown to provide additional cognitive and physical benefits [13,14]. Among the non-pharmacological interventions to reduce behavioral and psychological symptoms, sensory-based interventions have been developed, of which Snoezelen multisensory stimulation is certainly the most used so far, with positive effects on depression, anxiety and other BPS, despite its limited proven effect on cognition, functionality and quality of life [13]. Immersive virtual reality (VR), involving computer-generated three-dimensional environments or 360° videos combined with auditory stimuli such as music and/or ambient environmental sounds, represents another sensory-based approach used in patients with NCD to reduce anxiety, depression, apathy, and agitated behaviors [10,15,16,17,18,19]. VR-based reminiscence [20] therapy has been showed to favor older patients’ engagement in conversations and behaviors with their family and caregivers, which may further help reduce anxiety, apathy, and depression [16]. VR-based reminiscence therapy and VR-based cognitive rehabilitation have also been extensively used to stimulate cognitive functions [19,21,22]. A recent review showed that VR-based cognitive rehabilitation is effective at improving cognitive complaints, learning, memory skills, working memory, verbal fluency, spatial cognition, and instrumental activities of daily living in patients with mild NCD [23]. Compared to more traditional cognitive therapies, VR-based cognitive rehabilitation offers immersive and interactive environments that can simulate real-world scenarios in a controlled, customizable, and repeatable manner. By simultaneously stimulating multiple senses, virtual reality enhances patient motivation and engagement, which in turn improves adherence to therapy and may lead to greater cognitive benefits [24].

Despite these advantages, immersive VR also presents some limits that may restrain its usability as a therapeutic tool in nursing home residents. It implies the almost systematic use of a head-mounted display (HMD), which may not be well tolerated due to HMD heaviness or discomfort, blurry visual display, and a sense of isolation from the surrounding environment [15,25,26,27]. Also, the complexity of some hand controllers, used in conjunction with HMD to interact with the virtual environments, proved to be a problem for some residents [26,27].

A recent scoping review [26] on the use of immersive VR for promotion of health and well-being in people living in nursing homes concluded that immersive VR is most useful for residents when it provides opportunities for meaningful connection and engagement, when immersion is active rather than passive, and when it promotes reminiscence, socialization and discussion. Furthermore, as nursing home residents are an heterogeneous population, VR solutions that could meet different needs, offer a person-centered experience, a variety of contents and adapted solutions to interact with the environment should be developed [26,28,29]. Considering these recommendations, it is crucial to develop innovative immersive VR solutions that overcome the limitations of HMD, while providing realistic and personalized environments, interactive multisensory experiences, and opportunities for meaningful human-to-human interactions.

Cave automatic virtual environments (CAVE), which use projection-based VR on the walls of a room-sized cube, eliminate reliance on HMD and provide the possibility to move within the environment with a reduced risk of collision. They have also been employed with older adults to support fall-prevention therapy [30], train spatial abilities [31], improve cognitive and motor conditions [32] or reduce chronic musculoskeletal pain [33]. However, to our knowledge, only semi-immersive VR involving projections on one or two screens have been used to improve mood, engagement and induce reminiscence in nursing home residents [34,35,36]. Scarles et al. [36] compared immersive natural environments experienced through a VR-HMD and the same experience in a multisensory room using wall-projected virtual environments. The authors reported a clear preference of the participants for the wall-projected VR solution, which allowed them to be accompanied and share the experience. In contrast, the HMD solution was perceived as more isolating, underscoring the importance of social interaction and exchange for the participants.

A new type of VR room, developed by InMersiv Technologies (Brussels, Belgium), integrates computer-generated immersive realistic virtual environments projected onto four walls at 360°, accompanied by environmental sounds and airflow simulating wind. Patients can personalize the environments, be accompanied by a caregiver, and navigate the virtual space using a joystick. While this new immersive VR room represents a promising tool for delivering immersive and interactive experiences and overcoming some limitations associated with HMD, it is important to acknowledge that new kinds of immersive stimulation could also produce unintended effects. Previous studies have indeed shown that both over- and under-stimulation can exacerbate behavioral and psychological symptoms in nursing home residents [14,37]. For example, Moyle et al. (2018) [34] reported that VR experiences increased pleasure and alertness in people with dementia but also triggered fear and anxiety in some participants. A high level of immersion during VR may enhance emotional engagement, which can evoke unpleasant memories or emotions and lead to side effects such as sadness or disorientation [16,38]. Therefore, it is important to assess both positive and negative responses, and their repeatability over multiple sessions, before considering the VR room as a potential therapeutic tool.

Accordingly, this study aimed to investigate the tolerability and feasibility of a new VR room in nursing home residents. These outcomes were assessed through affective valence, perceived security, confinement, and fear using visual analog scales (VAS), along with verbal feedback. Sessions were recorded and analyzed using the Observed Emotion Rating Scale to assess positive and negative emotions, and the number of interactions was counted to evaluate active engagement. We hypothesized that immersion in the VR room would be well tolerated and feasible for most participants, and would promote positive affect and active engagement through its interactive, customizable features, and the presence of an accompanying person.

## 2. Materials and Methods

### 2.1. Study Design and Ethical Considerations

This feasibility study, using a one-arm mixed-methods design combining quantitative and qualitative data collection, assessed the feasibility of an immersive VR room featuring realistic, customizable environments projected on four walls and allowing active navigation for nursing home residents with neurocognitive and/or psychological disorders to enhance positive affect and engagement. It was conducted at La Cambre (Ter Kameren) nursing and care home in Brussels, Belgium. This feasibility study started after obtaining ethical approval from the Ethics Committee of Erasmus Hospital in March 2021 (B4062020000108). Before participation, all residents and their legal representatives were provided with detailed written and oral information about the study protocol. Informed consent was obtained from participants or their legal representatives.

### 2.2. Participants and Eligibility Criteria

Participants were eligible for inclusion if they were aged 60 years or older and had been diagnosed with a mild or major NCD, and/or a psychological disorder (e.g., depressive or anxiety disorder). The presence and classification of neurocognitive and psychological disorders were based on the diagnoses recorded in the patients’ medical files. Individuals were excluded if they had hearing or visual impairments that would prevent their participation, or if there was a risk of epilepsy or any other medical contraindication. A pre-selection process was carried out by the medical team, during which 40 residents were initially identified based on the inclusion and exclusion criteria. Of those, 20 residents were ultimately enrolled in the feasibility study. The most common reason for exclusion at this stage was the refusal of participation by the legal representative or the inability to contact the legal representative when the residents themselves were unable to provide consent.

### 2.3. Immersive VR Room

**Figure 1:**
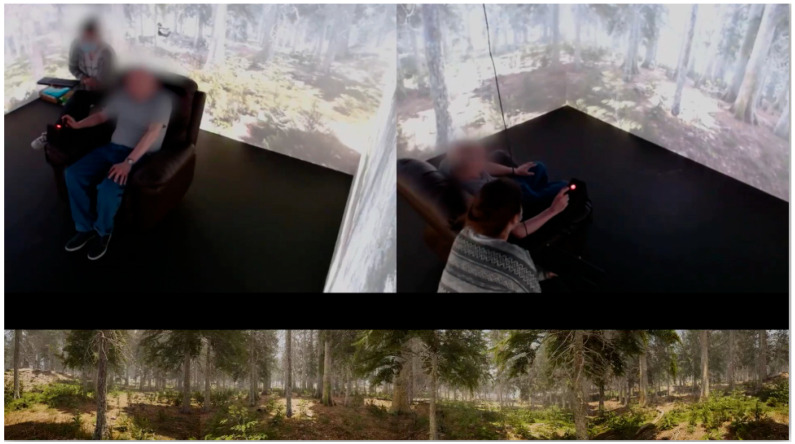
Immersive VR Room and participant setup. Participants were seated in a comfortable chair within the immersive VR room. The chair was equipped with a joystick (recognizable by the red), allowing virtual navigation within the environment. Each participant was accompanied by an occupational therapist during the session.

The VR setup developed by InMersiv Technologies (Brussels, Belgium) consists of a square immersive room measuring 3.6 m (11.8 feet) per side and 2.4 m (8 feet) in height. The immersive setup integrates visual and auditory stimuli. Each of the four walls operates as an independent projection surface, collectively forming a continuous 360° immersive 3D environment generated by four synchronized projectors. To improve immersion, high-definition computer-generated virtual environments are employed, ensuring the realistic appearance of landscapes, nature, animals, and objects, and allowing users to interact with these environments. During the sessions, participants, accompanied by an occupational therapist, were seated in a comfortable chair, equipped with a joystick, in the center of the VR room (Figure 1).

**Figure 2:**
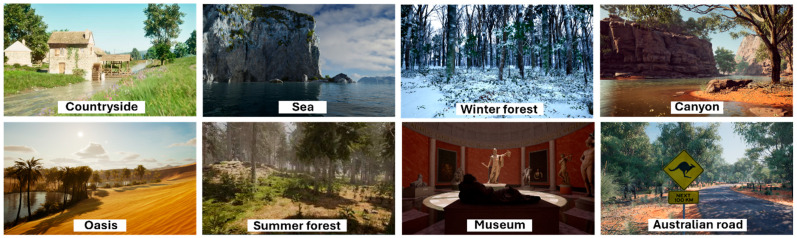
Immersive high-definition computer-generated realistic virtual environments.

Eight VR environments were used in this study: Countryside, Summer Forest, Winter Forest, Sea, Oasis, Canyon, Australian road and Museum (Figure 2). Participants were instructed to select the one they preferred for each session. They could explore the environment by turning their head or body. Additionally, the joystick enabled virtual navigation, allowing participants to move freely within the selected environment.

### 2.4. Protocol

The following data were collected from the patient’s medical records: sex, age, and diagnoses of neurocognitive and/or psychopathological disorders.

Baseline assessments were conducted prior to participation in the immersive sessions. They included an evaluation of cognitive functioning using the Mini-Mental State Examination (MMSE), a widely used and reliable screening tool of cognitive status in geriatrics [39], and behavioral disturbances and psychological symptoms were assessed using the Neuropsychiatric Inventory—Nursing Home Version (NPI-NH) [40,41]. This inventory was designed specifically for institutionalized individuals and captures the presence, frequency, and severity of behavioral and psychological symptoms based on caregiver reports. It includes 12 domains (delusions, hallucinations, aberrant motor behaviors, agitation/aggression, depression/dysphoria, anxiety, euphoria, apathy, disinhibition, irritability, sleep disturbances, and appetite/eating abnormalities), each rated for frequency and severity. The score for each domain is obtained by multiplying the frequency score by the corresponding intensity score.

Patients were then invited to take part in five immersive sessions in the VR room spread over 15 days. This session plan was intended to capture potential fluctuations in participants’ affective responses to the VR immersion, while remaining compatible with their daily routines and care schedules in the nursing home. The 15-day timeframe was considered sufficient to detect any delayed negative behavioral or affective effects.

For each immersive session, patients were accompanied from their room to the VR room by the occupational therapist. Prior to each session, participants rated their affective valence by responding to the question “How do you feel?” using a VAS. They were then invited to select one of the eight immersive environments.

The selected environment was then displayed, and patients remained immersed for a self-determined duration up to a maximum of 10 min. During the sessions, patients were encouraged to freely express their thoughts and emotions while engaging with the VR environment. They could also interact with the occupational therapist at any time. They were instructed to report any discomfort and could stop the session at any time if they felt psychologically or physically unwell. Otherwise, the session was terminated by the accompanying therapist if the maximum duration was reached. Each session was recorded using hidden cameras installed in the room. Patients’ reactions and comments were partially transcribed in real time by the examiner during the sessions, and the video recordings were subsequently reviewed to complete the initial transcriptions.

At the end of each session, patients rated their affective valence during the VR immersion and reported their perceived feelings of fear, confinement, and security using the VAS.

Following the session, participants responded to two open-ended questions, “What did you like?” and “What did you dislike?”, regarding the immersive experience. Oral responses were transcribed verbatim by the examiner onto an individual form for each session. Upon returning to their room, participants were asked to provide a last rating of the affective valence using the VAS.

To assess repeatability of the observations, four additional sessions were proposed over a 15-day period, each including the same VAS ratings and the two open-ended questions after the session. Patients or their legal representatives could refuse further participation at any time. At the start of each session, the patient could select an environment from the same list of eight available options.

Following the fifth session, a final interview was conducted with each participant to gather their impressions of the VR experience. Participants were encouraged to speak freely and spontaneously, without any predefined questions.

### 2.5. Analysis of Session Recordings

Two hidden cameras recorded each session for later analysis using the Observed Emotion Rating Scale (OERS), an observational tool with high validity and reliability, originally created to assess five emotional states in nursing home residents with dementia [42,43,44], and frequently used in studies involving patients with mild to major NCD [10,45,46]. This observational tool evaluates the presence and duration of two positive emotions (pleasure and interest) and three negative emotions (sadness, anxiety, and anger). Emotions were quantified over a 10 min observation period using a five-point time-based scale (1 = never; 2 = <16 s; 3 = 16–59 s; 4 = 1–5 min; 5 = >5 min).

These recordings also allowed for the identification of participants’ interactions with both the virtual environment and the caregiver, as well as the number of sessions (out of five) in which these interactions occurred. Video analyses and interaction reporting were conducted in a standardized manner for all participants. Interactions were coded retrospectively based on the video recordings, and notes taken during the sessions and subsequently compiled into a summary table.

### 2.6. Analysis of Verbatim

Verbatim responses collected to the questions “What did you like?” and “What did you dislike?” were subsequently subjected to a manual inductive thematic analysis [47]. The responses were first separated according to the question (aspects appreciated vs. not appreciated), then categorized into emerging themes and items through an iterative reading of the entire corpus. Themes and specific items were progressively developed by grouping verbatim statements expressing similar ideas until a stable and coherent thematic structure was established. For each item, the total number of occurrences was calculated across all participants and sessions, and detailed by virtual environment.

Spontaneous verbatim statements from patients during the five immersive sessions, as well as open-ended comments collected during the final interview, were transcribed into an Excel file. All transcripts were organized participant by participant, then subjected to a manual inductive thematic analysis [47]. Coding was carried out line by line: each statement was associated with an existing theme and subtheme, or led to the creation of a new theme and/or subtheme. This iterative process made it possible to group participants’ reports based on similarities in meaning and content. Themes and subthemes were discussed with the research team until consensus was reached, ensuring the validity of the final coding. The themes and subthemes, along with the corresponding verbatim reports, are presented in Table S1.

### 2.7. Statistical Analysis

We first assessed the distribution of the quantitative variables using the Shapiro–Wilk normality test. This test indicated that most variables did not follow Gaussian distribution. Consequently, results from the various assessments are presented as median [25th, 75th percentile], except for age and MSSE scores, and non-parametric statistical tests were used for analyses. A *p*-value of <0.05 was considered the threshold for statistical significance in all analyses.

The time course of VAS scores assessing affective valence (before, during, and after each session) was analyzed using the Friedman test for paired samples, considering both aggregated data across all sessions and data from each of the five sessions individually. A Friedman test was also conducted to examine changes across sessions for feelings of enclosure, fear, and security assessed using VAS, and for OERS scores. Since the OERS was originally developed for use in individuals with NCD, and because our sample included four participants with psychological disorders not associated with NCD, OERS analyses were conducted both including and excluding these four participants. When a significant main effect was found, Dunn’s post hoc test was performed to compare selected pairs of data. Statistical analysis was performed using GraphPad Prism 8.0 (GraphPad Software, San Diego, CA, USA).

## 3. Results

### 3.1. Participant Characteristics, Attendance Rate, and Selected Environments

Characteristics of the twenty participants initially included are presented in Table 1. Eleven participants were diagnosed with a major NCD (four with ethylic dementia with Korsakoff’s syndrome, one with vascular NCD, two with frontotemporal dementia, two with Alzheimer’s disease, and two with unspecified major NCD), and five were diagnosed with a mild NCD. Fourteen participants had a diagnosed psychological disorder (ten with chronic depression, two with depression and an anxiety disorder, and two with bipolar I disorder), either as an isolated condition (n = 3) or in association with an NCD (n = 10) or an intellectual disability (n = 1). Based on the domains of the neuropsychiatric inventory, the most frequent BPS with a severity score ≥ 2 (i.e., distressing to the patient) were depression/dysphoria and sleep disturbance (≥50% of participants), followed by anxiety and/or agitation (45%), irritability, aberrant motor behaviors, delusions and/or appetite and eating abnormalities (30%), apathy (25%), disinhibition (20%). The least frequent symptoms were euphoria and hallucinations (10%).

One participant with bipolar I disorder (major depressive episodes with psychotic features) and concomitant Alzheimer’s disease had to be excluded after two brief unsuccessful attempts. The participant’s restless behavior and mental preoccupation made it impossible to engage with the immersive experience. The remaining nineteen participants completed all five 10 min sessions and were included in the analyses and presentation of the results. Regarding the virtual environments selected by participants based on personal preference during each session, Countryside, Summer Forest, and Sea were chosen 19 times, Oasis was selected 15 times, Winter Forest 9 times, Canyon 7 times, Museum 4 times, and the Australian Road twice.

### 3.2. Affective Valence, Feelings of Fear, Security and Enclosure

**Figure 3:**
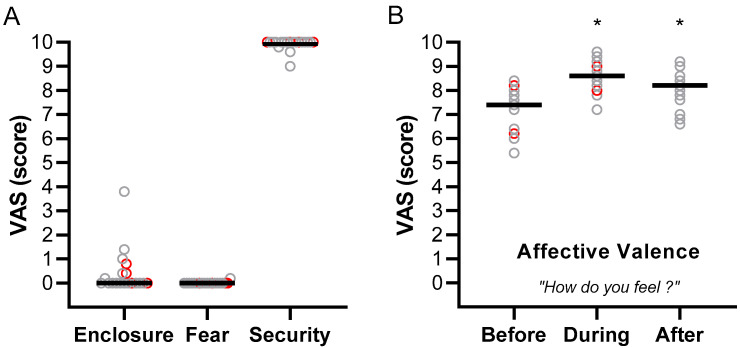
Visual analog scale (VAS) scores aggregated across all sessions for feelings of enclosure, fear, and security assessed during the VR immersion (**A**), and for affective valence assessed before, during, and after the sessions (**B**). Data for the nineteen participants who completed all sessions are shown as individual values, with the median indicated by a horizontal line. Grey circles represent participants with NCD, whereas red circles represent participants without NCD. * *p* < 0.05 compared to before immersion.

Figure 3 presents the aggregated results from all sessions for the VAS scores assessing perceived feelings of enclosure, fear, and security during immersion in the VR room (Figure 3A), as well as the affective valence assessed before, during, and after the sessions (Figure 3B).

Except for a few participants, VAS scores for enclosure and fear were close to 0, while scores for the feeling of security were close to 10 (Figure 3A). There was no significant difference between sessions (Friedman test; *p*-values ranging from 0.149 to 0.406; see detailed results in Figure S1A).

Affective valence was assessed by asking participants “How do you feel?” before, during, and after each VR session. When data from all sessions were aggregated (Figure 3B), affective valence improved significantly during the immersion in the VR room compared to before the immersion (Dunn’s post hoc test before vs. during, *p* < 0.001) and remained higher after the VR session (Dunn’s post hoc test before vs. after, *p* = 0.010). When the analysis was performed separately for each of the five sessions, the increase observed during immersion was significant in all sessions (*p*-values ranging from <0.001 to 0.014), whereas post-session scores remained significantly higher than pre-session values only in the fourth and fifth sessions (*p* = 0.008 and *p* = 0.045, respectively; see detailed results in Figure S1B).

### 3.3. Affects and Interactions Observed During VR Sessions

#### 3.3.1. Observed Emotion Rating Scale

**Figure 4:**
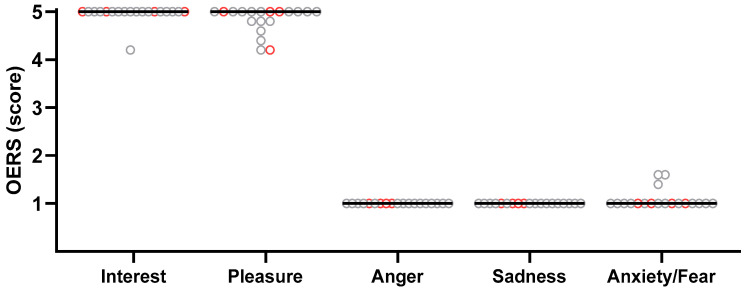
Observed Emotion Rating Scale (OERS) scores aggregated across all sessions for Interest, Pleasure, Anger, Sadness, and Anxiety/Fear during VR immersion. Data for the nineteen participants who completed all sessions are shown as individual values, with the median indicated by a horizontal line. Grey circles represent participants with NCD, whereas red circles represent participants without NCD.

The OERS was used to quantify the presence and duration of two positive affects (pleasure and interest) and three negative affects (sadness, anxiety, and anger). Figure 4 presents the results aggregated across the five sessions, with subjects with NCD identified by grey circles and those without NCD by red circles. The scores indicate the time spent displaying each affect during the 10 min immersion (1 = never; 2 = <16 s; 3 = 16–59 s; 4 = 1–5 min; 5 = >5 min).

All participants except one exhibited interest for at least 5 min during immersion. Twelve participants (nine with NCD) displayed pleasure for more than 5 min, while seven (six with NCD) did so for slightly less than 5 min. Regarding negative affects, none of the participants exhibited anger or sadness, and only three (all with NCD) showed expressions of anxiety or fear during a few seconds. Statistical comparisons across the five sessions revealed no significant differences for any of the affects, whether analyses included all participants (n = 19; *p*-values ranging from 0.070 to 0.99) or were restricted to participants with NCD (n = 15; *p*-values ranging from 0.161 to 0.99; see detailed results in Figure S2).

#### 3.3.2. Interactions During VR Sessions

The types of observed interactions included (1) visual exploration of the environment, corresponding to the active observation of various elements within the VR environment; (2) reactions to visual elements of the environment, when the participant showed verbal/physical reactions to aspects of the VR environment; (3) active navigation within the environment using the joystick, when the participant moved around and actively explored the virtual space; (4) interactions with the accompanying person, when the participant addressed the caregiver; and (5) reactions to sounds, when the participant reacted to background sounds or music, for example by commenting, humming, or expressing an emotion related to the sound. Table 2 presents the number of sessions per participant during which each interaction was observed, and the number of participants who demonstrated the interactions during one or more sessions.

### 3.4. Qualitative Analysis of Verbatim Responses to Questions

Table 3 presents participants’ responses to the guided questions regarding aspects of the immersive experience they liked and disliked. Responses were grouped into main themes reflecting the most frequently mentioned elements. For each theme, the specific items cited by participants, the number of reports and participants, and environment in which they occurred are also reported. Regarding the liked aspects, the most frequently cited were visual elements of the nature VR environments (43 reports in total), followed by soundscapes, either chosen by the participant or associated with the environment (36 reports); aesthetic and realistic attributes of the environments, including nature, landscapes, and sky (27 reports); possibilities offered by the technology (5 reports); and the soothing atmosphere (4 reports). For the disliked aspects, the most frequent were elements related to fauna; some participants reported too many animals and animal sounds, whereas others reported the opposite (9 reports). Navigation within the environment was reported as being difficult 4 times by one participant. Two participants perceived cliffs in the Sea environment as oppressive, and one participant reported twice a lack of realism.

### 3.5. Qualitative Analysis of Spontaneous Verbatim from Sessions and the Final Interview

**Figure 5:**
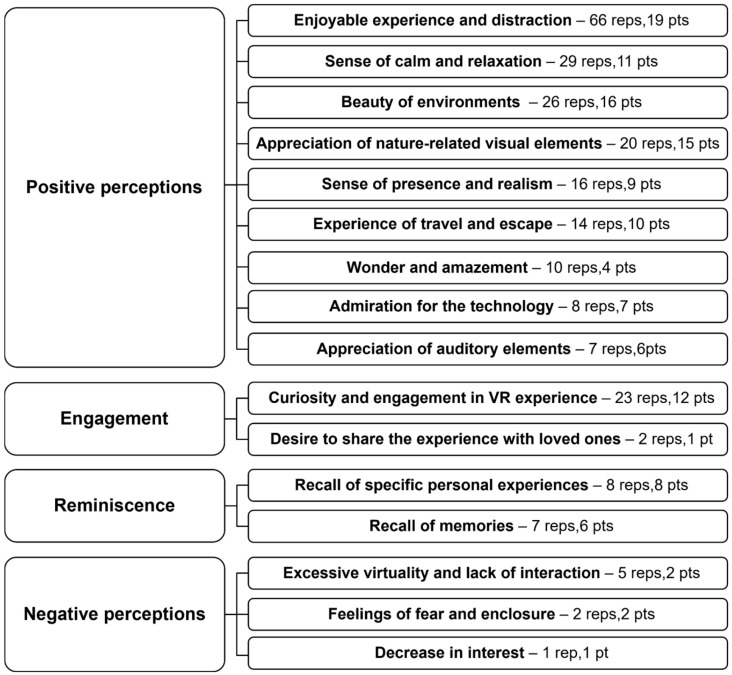
Main themes and subthemes, along with the total number of reports (reps) and participants (pts) contributing them across the five immersive sessions and the final interview.

Spontaneous verbatim statements from patients during the five immersive sessions, along with open-ended comments collected in the final interview, were organized by main themes and subthemes during the thematic analysis process (Figure 5). The verbatim reports corresponding to each theme and subtheme are detailed in Table S1.

Four main themes were defined: “Positive Perceptions”, “Engagement”, “Reminiscence experience”, and “Negative Perceptions”. Nine subthemes were identified under the main theme Positive Perceptions: “Enjoyable emotional experience and distraction”, “Sense of calm and relaxation”, “Beauty of environments”, “Appreciation of nature-related visual elements”, “Sense of presence and realism”, “Experience of travel and escape”, “Wonder and amazement”, “Admiration for the technology”, and “Appreciation of auditory elements”. Two subthemes were identified under the theme Engagement: “Curiosity and engagement in the VR experience”, and “Desire to share the experience with loved ones”. Two subthemes were identified under Reminiscence: “Recall of specific personal experiences” and “Recall of memories”. Finally, three subthemes were identified under Negative Perceptions: “Excessive virtuality and lack of interactions”, “Feelings of fear and enclosure”, and “Decrease in interest”. For each subtheme within the main themes, the total number of reports (reps) and the number of participants (pts) who contributed these reports are summarized in Figure 5, ordered by report frequency.

For the main theme “Positive Perceptions”, the most frequently cited reports (66 in total across 19 participants) were related to the subtheme “Enjoyable experience and distraction” provided by the sessions in the VR room (Excerpts: *“I had a great time. It felt good.” [P1]; “It really makes me happy.” [P5]; “It was beautiful and satisfying.” [P13]; “It helps to clear your mind and escape the everyday.” [P9]*). The subtheme “Sense of calm and relaxation” was mentioned in 29 reports by 11 participants (Excerpts: *“It’s very relaxing, I’m very calm.” [P20]; “I feel relaxed despite my pain.” [P7]; “It’s an enjoyable and relaxing activity.” [P10]*), while “Beauty of environments” was highlighted 26 times by 16 participants (Excerpts: *“At least here I see beautiful things.” [P1]; “It’s really pretty.” [P9]; “The landscape is impressive.” [P2]*). “Appreciation of nature-related visual elements” appeared in 20 reports from 15 participants (Excerpts: *“I like the sand.” [P16]; “I liked seeing the animals.” [P20]; “The rocks at the water’s edge are nice.” [P7]*), “Sense of presence and realism” was reported 16 times by 9 participants (Excerpts: *“It’s as if I were there.” [P13]; “It feels like we’re in Africa.” [P15]; It’s a very realistic environment.” [P20]*). “Experience of travel and escape” was reported 14 times by 10 participants (Excerpts: *“I travelled to the sea.” [P10]; “It’s really nice, you feel completely transported.” [P7]; “It allows you to travel.” [P6]*). “Wonder and amazement” was expressed 10 times by 4 participants (Excerpts: *“My God, that’s amazing.” [P8]; “Wow, I wasn’t expecting that!” [P9]; “It’s unique” [P19]*). “Admiration for the technology” appeared in 8 reports by 7 participants (Excerpts: *“It’s impressive what technology allows, nonetheless, it’s crazy.” [P2]; “Impressed by the setup.” [P8]; “Technology is amazing.” [P13]*), and “Appreciation of auditory elements” was mentioned 7 times by 6 participants (Excerpts: *“I hear the sound of the waves.” [P3]; “The bird is happy, it sings all the time.” [P13]; “I enjoyed the music.” [P4]*).

Regarding the theme “Engagement,” reports related to the subtheme “Curiosity and engagement in VR experience” were mentioned 23 times by 12 participants (Excerpts: *“I liked everything, I want to come back.” P3]; “I’m curious, so I like it.” [P15]; “It’s interesting, it makes me want to discover the next environments.” [P20]*). The “Desire to share the experience with loved ones” was expressed twice by one participant (Excerpts: *“When I tell my granddaughter, she’ll want to come too.” [P9]*).

For the main theme Reminiscence, “Recall of memories” was mentioned 7 times by 6 participants (Excerpts: *“It reminds me of memories.” [P5]; “It reminded me of good memories.” [P16]; “This reminds me of memories and keeps me occupied.” [P9]*), and “Recall of specific personal experiences” was made 8 times by 8 participants (Excerpts: *“I felt rejuvenated, it reminds me of my youth.” [P18]; “I traveled to this place.” [P16]; I like the forest, because when I was younger, we often went for walks in the forest with my father. And I liked it.” [P1]*)

Finally, under the theme Negative Perceptions, “Excessive virtuality and lack of interaction” was reported 5 times by 2 patients (Excerpts: *“I would have liked more interactions.” [P6]; “It’s pretty, but very virtual*.” *[P4]*). “Feelings of fear and enclosure” were expressed 2 times by 2 participants (Excerpt: *“It’s too close, it scares me.” [P3]*)*,* and a “Decrease of interest” was evoked once by 1 participant (Excerpt: *“Tired.” [P1]*).

## 4. Discussion

While the feasibility of VR delivered through HMD has been explored in nursing home residents, the feasibility of a VR room projecting immersive and interactive environments at 360° across four surrounding walls has not yet been evaluated. The objective of the present study was therefore to explore the tolerability and feasibility of immersion in this type of VR room in nursing home residents with neurocognitive and psychological disorders. To that end, twenty residents were invited to participate in five 10 min immersive sessions in virtual environments of their choice, which they tailored to their preferences and navigated actively using a joystick.

Our findings indicate that immersion in the VR room was feasible and well tolerated by all participants, except for one individual with bipolar I disorder and concomitant Alzheimer’s disease, who was excluded after two unsuccessful attempts. VAS scores indicated that most participants felt secure and reported little to no fear during immersion. Furthermore, positive affect increased during immersion compared to pre-immersion levels. This effect was maintained beyond the immersion from the fourth session. OERS scores showed that most participants expressed, either verbally or through nonverbal behavior, pleasure and interest for more than five minutes. Negative effects were rare, with only a few residents exhibiting mild feelings of confinement or brief episodes of fear or anxiety lasting only a few seconds. In addition, numerous interactions with the virtual environments and with the accompanying person were observed during the sessions. Verbal reports collected during the sessions and the final interview, along with responses to close-ended questions, indicated that immersion in the VR room was an emotionally positive and relaxing experience. Participants particularly appreciated the aesthetics and realism of the environments, as well as the presence of the diverse nature-related visual and auditory elements. The experience enabled participants to travel virtually and to recall personal memories. Engagement was high, as reflected by frequent interactions and the participants’ willingness to repeat the experience, explore new environments, and share it with loved ones. No negative side effects were reported, although a few participants mentioned difficulties navigating the environments, feelings of fear or enclosure, lack of realism or interactivity, or decreased interest in continuing the experience.

### 4.1. Feasibility and Tolerability of Immersion in the VR Room

Based on the “Reduced stress threshold” model in patients with major NCD [48], multiple and/or unknown stimuli could produce stress responses and strengthen some symptoms. Negative emotions (e.g., anxiety, fear…) have indeed been reported on several occasions in elderly users during VR experiences [34,45,49,50]. Therefore, an important concern was to determine whether the visual and auditory stimuli delivered within the VR room might be overwhelming for residents with cognitive impairments and BPS, potentially triggering negative emotional reactions or other symptoms. However, our findings indicate that the experience was feasible and well-tolerated by patients with NCD and psychological symptoms, with the exception of one patient diagnosed with bipolar I disorder and Alzheimer’s disease. This resident presented a particularly complex symptom profile (i.e., marked behavioral disturbances, a strong need to wander, delusional ideas, persistent mental preoccupation, and pronounced anxiety when familiar landmarks are absent) which appeared incompatible with initiating new activities in unfamiliar settings. All other residents completed all the sessions. The presence of an accompanying person in the VR room, along with the ability to select and customize the environment according to personal preferences, likely contributed to making the experience both reassuring and enjoyable.

These observations are consistent with studies using VR-HMD to enhance positive affect in nursing home residents with mild to major NCD, which generally reported no side effects or, in a small subset of patients, issues such as cybersickness-related symptoms (e.g., nausea, dizziness, disorientation, confusion) or discomfort caused by the HMD [10,15,25,51]. Although cybersickness was not directly assessed in the VR room using a dedicated questionnaire, no participants requested to discontinue the immersion due to symptoms, nor did they report such symptoms during the interviews. The absence of reported cybersickness-like symptoms may be partly attributable to the use of a CAVE-type VR room, rather than an HMD, which has been shown to induce less cybersickness [52]. Additional factors may include user-controlled navigation via a joystick and the older age of participants, as susceptibility to VR sickness seems to decline with age [53]. We also cannot rule out the possibility that the 10 min session duration was too short to elicit cybersickness, since shorter VR exposures are generally associated with a lower risk of such symptoms compared to sessions exceeding 10 min [25,54].

### 4.2. Effects on Affective Valence and Engagement

Institutionalized older adults frequently feel anxious, lonely and confused due to a loss of autonomy and freedom, and lack of meaningful social interactions, which exacerbate cognitive decline and is a major risk factor for depression [9,55]. Providing residents with the opportunity to have pleasant experiences in natural or familiar environments outside the nursing home could be beneficial to mitigate these issues [15,38]. However, access to such activities may be limited by residents’ physical or cognitive impairments, or by the distance to suitable environments. Offering immersive experiences in safe, customizable settings that residents can no longer visit may therefore provide a valuable non-pharmacological approach to relive pleasant past events, evoke positive emotions and relaxation, and enhance engagement. In this context, immersive VR has been demonstrated to be a viable alternative to outdoor activities. However, the systematic reliance on HMDs and hand controllers limits its use in part of elderly residents and reduces opportunities for human-to-human interactions. Therefore, the present study investigated the feasibility of immersive experiences in the new VR room and their immediate effects on affective valence and engagement, a system that eliminates the need for an HMD, reduces collision risk, allows interactive, customizable VR environments, and can be shared with an accompanying person. Affective valence increased significantly during the immersive sessions and remained elevated compared to pre-immersion levels from the fourth session onward, suggesting that repeated experiences may produce more consistent benefits. Most participants displayed verbal and nonverbal expressions of pleasure and interest for over five minutes, while anger, fear, and sadness were rarely observed. Engagement in the activity was high, as reflected by frequent interactions with the accompanying person and reactions to visual and auditory stimuli within the virtual environments, as well as active involvement through navigation within the VR space. Reports collected during sessions and interviews highlighted that the VR room was perceived as emotionally positive, with many participants describing the experience as enjoyable and relaxing, allowing them to explore freely beautiful virtual environments. They also reported a strong sense of presence and realism, particularly when visiting places that evoked personal memories. Engagement was further manifested by requests to repeat the experience and explore new environments. These results thus support the feasibility of using the VR room to improve affective valence and stimulate engagement in residents with NCD and psychological disorders. 

Our results are in line with prior findings showing improved mood states and engagement when using immerse VR with HMD [15,49,56,57] or semi-immersive VR with one or two large screens [34,35,58] in institutionalized older adults with mild to major cognitive impairments. The possibility to customize the virtual environments, to navigate the environments using a simple joystick, and to directly share the experience with the accompanying person within the VR room likely enhanced positive emotions and increased engagement [15,59].

### 4.3. Limitations

The present study is not a clinical trial but a single-arm feasibility study assessing the tolerability and feasibility of using a new type of VR room in nursing home residents. It includes a small sample size, short-term follow-up, has no control group, and participants were recruited from a single nursing home. Caution is therefore warranted regarding the generalizability of these findings, and a multicentric randomized controlled trial with a longer follow-up period is required to confirm the VR room’s potential as a non-pharmacological therapeutic intervention.

Secondly, the OERS was chosen in the present study to assess positive and negative affects during the VR sessions because of its strong psychometric properties, including demonstrated validity and reliability tested on large samples [43,44]. However, like most emotion observational tools [43], it was initially developed and validated with samples of patients with dementia. Although it has rarely been used in studies involving more diverse samples [60], we acknowledge that applying it to a sample partly composed of residents without NCD (four participants out of the final sample of nineteen) represents a limitation and warrants caution when interpreting these findings. Furthermore, given the small sample size, results from assessments such as the OERS and VAS should be interpreted with caution. Nevertheless, the convergence of quantitative data (VAS, OERS, and the number of interactions with the environments and caregiver) and qualitative data (participants’ responses to directed questions and spontaneous verbatim), which indicated interest, engagement, numerous positive emotions, and rare reports of negative perceptions, supports our confidence that OERS and VAS reflected real changes in affect.

Thirdly, side effects (e.g., cybersickness) and impacts on mood were not assessed using dedicated tools, representing another limitation. However, given the challenges patients with NCD may face in comprehension and sustained attention, the primary aim of this study was to reduce the quantitative burden and allow sufficient time for interviews. Future studies could specifically examine these aspects to gain a more comprehensive understanding of both the positive and negative effects of this type of VR setup.

Finally, the impact of the VR environments’ theme and content was not directly assessed in present study, although it is a potential modulator of emotional responses and engagement. The aim of this study was to evaluate the tolerability and emotional response to the VR room itself. Consequently, we relied on existing literature and the environments available in the system to select those proposed in the project. Previous research indicates that customizable environments replicating natural scenery and selected according to personal preferences are most appropriate for patients with NCD to positively influence emotions, promote autonomy and engagement [11,38,49,51,61,62]. Accordingly, the environments offered were mainly natural sceneries, with the exception of the museum, which was selected only four times. During each session, participants chose the environment they wished to visit from the available options. For this reason, not all participants tested all environments but only their preferred ones. This self-selection may have introduced subjective bias and resulted in large differences in the number of participants choosing each environment, thereby compromising the reliability of inter-environment comparisons.

### 4.4. VR Room Applications: Advantages, Limits, and Perspectives

Although VR immersion through an HMD was shown to be feasible in older patients with NCD and BPS, most of the studies encountered difficulties with HMD in terms of fitting the device over glasses, blurred vision, physical discomfort due to heaviness of some HMD, and difficulties interacting with the environment using hand-controllers [15,27,49,57,63]. Some elderly users also found the experience with the HMD intrusive and felt a lack of social connectedness [27]. Additionally, the use of HMDs and hand controllers can pose safety risks for older adults, including collisions and falls [26,27]. Alternative solutions are therefore needed to accommodate older adults’ physical and visual limitations while supporting meaningful social interaction and safe, unrestricted movements.

The VR room mitigates many of the inconveniences and risks linked to HMDs and hand controllers. In this setting, patients are accompanied, enabling shared experiences and support in customizing and navigating the virtual environments. Navigation was facilitated by a simple analog joystick that can be grasped with the whole hand, making it suitable for individuals with impaired fine motor skills. Patients can move freely within the VR room while maintaining full awareness of their own body and the surrounding environment. Although the present setup lacked ceiling and floor projection, likely reducing the level of immersion, this may also have advantages. Matsangidou et al. [11] reported that the high immersion provided by HMDs can increase disorientation in patients with dementia, as some participants were unable to distinguish between the virtual and physical environments. Similarly, Li et al. [24] found that VR-based cognitive interventions using semi-immersive systems were more effective across multiple cognitive outcomes than both non-immersive and fully immersive VR. The authors suggested that semi-immersive VR may achieve an optimal balance between engagement and cognitive load, maximizing involvement while minimizing adverse effects, making it a promising approach for cognitive rehabilitation in individuals with NCD.

From a practical standpoint, the improvements offered by the proposed VR room are consistent with recommendations from previous authors, who emphasize the importance of aligning the technology with the preferences and capabilities of older adults, increasing interactivity, providing customizable, realistic and relaxing nature-inspired environments, and enabling multi-user experiences to foster social interaction [11,34,49,51,64,65,66,67]. This is particularly relevant because, as neurocognitive impairments progress and memory and communication abilities decline, opportunities for meaningful shared interactions become increasingly limited [64]. By fostering reminiscence, social interaction and engagement, immersion in the VR room may also serve as a tool to stimulate cognitive functioning.

Older adults with NCD also face an elevated fall risk during daily activities that require the simultaneous performance of physical and cognitive tasks under a dual-task paradigm. In this context, VR has emerged as an effective intervention to enhance motor–cognitive integration [68]. Thanks to its advantages over HMD-based VR (e.g., greater freedom of movement, possibility of physical interaction with other users, and integration of a motion-tracking system in the new version of the VR room enabling interaction with the environment), the VR room could support the development of novel motor–cognitive dual-task VR training protocols. It also offers the practical possibility of organizing multimodal interventions, which typically combine physical and cognitive training, social activities, and psychological support. Such multimodal interventions produce significantly greater improvements in global cognition, attention, and executive function than single-modal interventions in older adults with NCD [69].

However, before evaluating the potential of the VR room as an approach to stimulate cognitive functioning, it was essential to assess its tolerability and emotional impact, as these factors are prerequisites for establishing the VR room as a sustainable intervention in nursing homes [70]. Based on our findings, the 10 min immersion sessions featuring predominantly nature-based environments were well tolerated and may represent a safe starting point to minimize VR-related side effects during initial exposure while maximizing potential benefits [53].

This feasibility study focused on assessing tolerability, affective responses and engagement during immersion in the VR room among patients with neurocognitive and psychological disorders, using nature-inspired environments and a one-on-one format with a caregiver. To further explore the potential role of social interaction in the VR room on affective responses and cognitive engagement, future studies could involve family members or other residents. Another promising direction would be to expand the variety of environments and examine how environmental characteristics influence affective valence and cognitive engagement using mixed-methods approaches. Applying this work in different nursing home settings, with larger samples and a broader range of neurocognitive and psychological disorders, would help better determine the most appropriate parameters for each target population and therapeutic objective.

Despite the promising benefits offered by the VR room, implementing and integrating this type of VR setup in nursing homes also presents practical challenges, including the need for a dedicated, adequately sized space and higher costs compared to HMDs. Moreover, as with other VR systems, ongoing hardware and software maintenance, staff adherence and training, and the development of features to enhance interactivity and diversify applications will need to be addressed to develop this VR technology into a sustainable, accessible, and effective non-pharmacological intervention.

## 5. Conclusions

Immersion in the VR room was feasible and well-tolerated for nearly all participants. Only one individual, with bipolar I disorder and Alzheimer’s disease, was unable to engage due to pronounced restlessness and mental preoccupation. Most participants felt secure and reported minimal fear or confinement. Affective valence improved during immersion and remained elevated shortly after the session in the final two sessions. Observational and self-reported data showed sustained pleasure and interest, as well as frequent interactions with both the virtual environments and the accompanying person, indicating strong engagement. The VR experience was perceived as emotionally positive, relaxing, and aesthetically pleasing, and induced reminiscence. No adverse effects were observed, though minor issues such as navigation difficulties or excessive virtuality were reported by a few participants. Overall, the experience in the VR room enhanced positive affect, provided a highly engaging experience and fostered social interactions. Since the present research is a monocentric feasibility study with a small sample size and short-term follow-up, the findings should be considered preliminary. The potential of the VR room as a therapeutic approach needs to be confirmed in future controlled, multicentric studies with longer follow-up periods.

## Figures and Tables

**Table 1:** Participant characteristics.

**Total Sample, n**	**20**
Female, n	10
Male, n	10
Age, years, mean ± SD	75.1 ± 7.9
MMSE score, mean ± SD	21.0 ± 6.6
**Clinical diagnosis, n**	
Major NCD, n	11
Mild NCD, n	5
NCD without psychological disorder, n	6
NCD with psychological disorder, n	10
Psychological disorder only, n	3
Psychological disorder with an intellectual disability, n	1
**NPI-NH domain scores, median [25th; 75th percentiles]**	
Depression/Dysphoria	2.5 [0.3; 6.0]
Sleep and night-time behavior disturbance	2.5 [0.0; 8.0]
Anxiety	2.5 [0.0; 7.5]
Agitation/aggression	1.5 [0.0; 6.0]
Irritability	0.5 [0.0; 4.0]
Aberrant motor behaviors	0.0 [0.0; 5.5]
Delusions	0.0 [0.0; 5.5]
Appetite/eating abnormalities	1.0 [0.0; 3.8]
Apathy	0.5 [0.0; 2.8]
Disinhibition	0.0 [0.0; 0.8]
Euphoria	0.0 [0.0; 0.8]
Hallucinations	0.0 [0.0; 0.0]
Patients with severity score for NPI-NH domain ≥ 2 *, n (%)	
Depression/Dysphoria	11 (55)
Sleep and night-time behavior disturbance	10 (50)
Anxiety	9 (45)
Agitation/aggression	9 (45)
Irritability	6 (30)
Aberrant motor behaviors	6 (30)
Delusions	6 (30)
Appetite/eating abnormalities	6 (30)
Apathy	5 (25)
Disinhibition	4 (20)
Euphoria	2 (10)
Hallucinations	2 (10)

NCD, neurocognitive disorder. NPI-NH, neuropsychiatric inventory—Nursing Home Version. * A severity score of 2 indicates a moderate symptom, which is distressing to the patient but can be redirected by the caregiver.

**Table 2:** Interactions observed during the sessions in the VR room: type of interaction, number of sessions per participant, and number of participants who exhibited the interaction.

Type of Interaction	Number of Sessions per Participant				Number of Participants (Out of 19)
	Median	P25	P75	Range	
Visual exploration of the VR environment	5	5	5	5–5	19
Reactions to visual elements of the VR environment	5	3	5	0–5	17
Navigation within the VR environment	5	3	5	0–5	17
Interactions with caregiver	4	3	5	0–5	18
Reactions to VR environment sounds/music	3	1.5	4	0–5	16

P25, percentile 25. P75, percentile 75.

**Table 3:** Summary of participants’ responses to directed questions regarding appreciated and non-appreciated features during immersive sessions in the VR room.

Theme	Representative Items	Number of Reports and Participants	Environments (n Reports)
**What did you like during the immersion?**			
**Visual elements**	Presence of rocky landscape and cliffs	4; 4	Sea (3), Canyon (1)
	Aquatic features (water, sea and lake)	10; 6	Countryside (3), Oasis (3) Summer Forest (2), Sea (1), Canyon (1)
	Presence of fauna (birds, seagulls, ducks, rabbits, squirrels, corrals, and fishes)	15; 9	Sea (7), Summer Forest (3), Winter Forest (2), Countryside (2), Oasis (1)
	Vegetation diversity (plans, flowers, trees, palm trees, details of the vegetation, richness of the greenery, and plants by the water)	14; 9	Summer Forest (5), Countryside (4), Oasis (3), Winter Forest (1), Canyon (1)
**Soundscapes**	Musical background (chosen by the subject)	15; 8	Winter Forest (4), Oasis (4), Countryside (3), Summer Forest (2), Sea (2)
	Bird sounds	13; 8	Summer Forest (6), Sea (2), Countryside (2), Oasis (1), Australian road (1)
	Nature ambient sounds (sea waves and wind)	8; 7	Sea (6), Summer Forest (1), Winter Forest (1)
**Aesthetic and** **realistic** **attributes**	Beautiful images in general	10; 8	Sea (3), Summer Forest (2), Countryside (2), Museum (2), Oasis (1)
	Realism of the environments (sand moving with the wind, feeling of floating on the water, all is very realistic, it looks like reality…)	6; 5	Oasis (2) Summer Forest (2), Sea (2)
	Beauty of the nature	6; 4	Countryside (2), Summer Forest (1), Winter Forest (1), Oasis (1), Canyon (1)
	Beauty of landscapes and depth of the visual field	3; 2	Oasis (3)
	Beauty of the sky (clear blue sky and sunlight)	2; 2	Sea (1), Countryside (1)
**Possibilities** **offered** **by the** **technology**	Freedom of movement (climb the rocks, dive and swim in water, possibility to interact with environments, to travel where I want…)	4; 3	Oasis (2), Countryside (1), Sea (1)
	Visit nature virtually without having to travel	1; 1	Summer Forest (1)
**Soothing** **environment**	Winter forest atmosphere	1; 1	Winter Forest (1)
	Calming ambiance	3; 3	Winter Forest (1), Sea (1), Museum (1)
**Was there anything you dislike during the immersion?**			
**Fauna**	Too many animals (there are too many bird sounds, too many animals, it feels unnatural, animal noises are disturbing…)	3; 3	Summer Forest (1), Countryside (1), Canyon (1)
	Too few animals (lack of animals, would like more animals, more birds, not only to hear the birds but also see them, lack of birds and ducks…)	6; 5	Summer Forest (2), Sea (2), Canyon (1), Australian Road (1)
**Navigating** **within the** **environments**	Movements are difficult, navigating the environments is too slow, navigating is difficult and slow, I would like to be able to catch things…)	4; 1	Countryside (1), Oasis (1), Sea (1), Winter Forest (1)
**Lack of realism**	Mountains are not very realistic, having too many sounds makes it seems unnatural	2; 1	Summer Forest (1), Countryside (1)
**Elements** **perceived as** **oppressive**	Would feel more comfortable with an open sea without rocks, feeling of oppression between the cliffs and the water	2; 2	Sea (2)

## Data Availability

The data presented in this study are available from the corresponding author upon reasonable request.

## Supplementary Materials

The following supporting information can be downloaded at https://www.mdpi.com/article/10.3390/healthcare14050588/s1. Table S1: Verbatim reports organized by themes (main themes highlighted in grey) and subthemes (left column); Figure S1: Visual Analog Scale (VAS) scores across the five sessions (S1–S5) for feelings of enclosure, fear, and security assessed during VR immersion, and for affective valence assessed before, during, and after each session; Figure S2: Observed Emotion Rating Scale (OERS) scores across sessions 1 to 5 (S1–S5) for Interest, Pleasure, Anger, Sadness, and Anxiety/Fear during VR immersion.

## Abbreviations

The following abbreviations are used in this manuscript:

BPS	Behavioral and psychological symptoms
CAVE	Cave automatic virtual environment
HMD	Head-mounted display
MMSE	Mini-Mental State Examination
NCD	Neurocognitive disorders
NPI-NH	Neuropsychiatric Inventory—Nursing Home Version
OERS	Observed Emotion Rating Scale
VAS	Visual analogue scale
VR	Virtual reality
