# Supplementary material for: An Immersive Virtual Reality Room to Enhance Positive Affect and Engagement in Nursing Home Residents with Neurocognitive and Psychological Disorders: A Feasibility Study
Source: Healthcare (Basel). 2026 Feb 26;14(5):588. doi: 10.3390/healthcare14050588 (PMC12984680; doi:10.3390/healthcare14050588)
Supplement: Supplementary file 1 [file healthcare-14-00588-s001.zip › healthcare-4149225-supplementary.pdf]

Supplementary materials

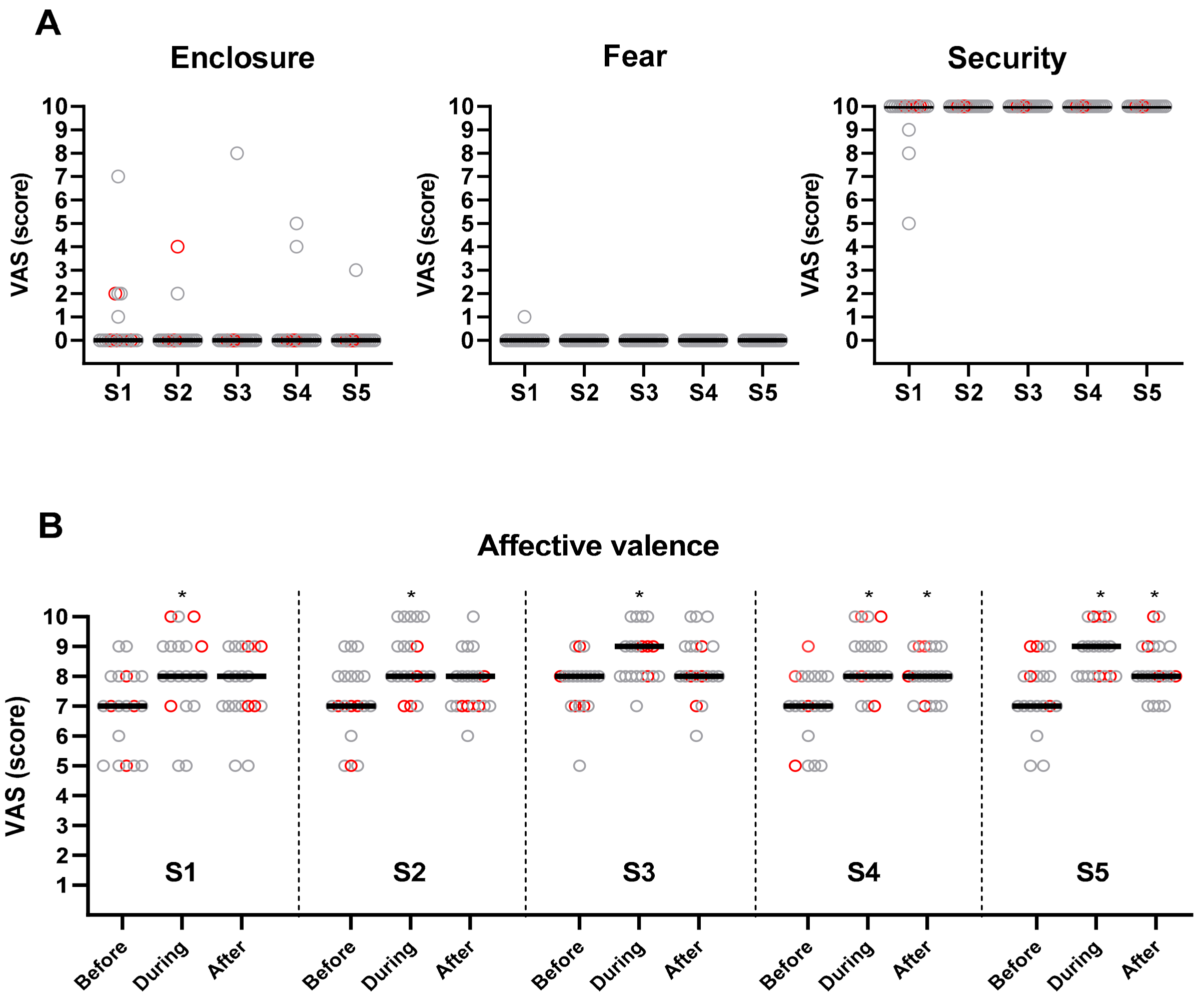

Figure S1. Visual Analog Scale (VAS) scores across the five sessions (S1–S5) for feelings of enclosure, fear, and security assessed during VR immersion (A), and for affective valence assessed before, during, and after each session (B). Data for the nineteen participants who completed all sessions are shown as individual values, with the median indicated by a horizontal line. Grey circles represent participants with NCD, whereas red circles represent participants without NCD. *$p<0.05$ compared to before immersion.

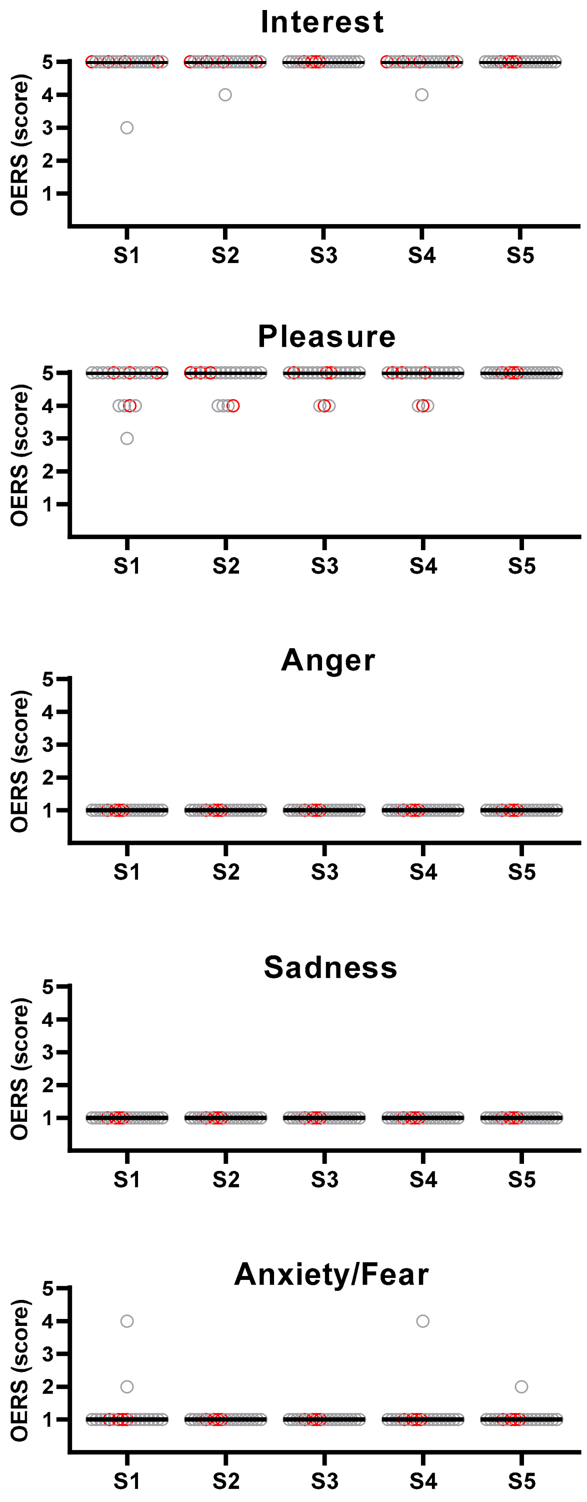

Figure S2. Observed Emotion Rating Scale (OERS) scores across sessions 1 to 5 (S1–S5) for Interest, Pleasure, Anger, Sadness, and Anxiety/Fear during VR immersion. Data for the nineteen participants who completed all sessions are shown as individual values, with the median indicated by a horizontal line. Grey circles represent participants with NCD, whereas red circles represent participants without NCD.

Table S1. Verbatim reports organized by themes (main themes highlighted in grey) and subthemes (left column).

Subthemes	Verbatims
	**Positive perceptions**
**Enjoyable experience and distraction**	"It was good, I liked it, it's entertaining." – Participant 15, Session 4, Australian Road
	"I like it, it's pleasant." – Participant 6, Session 5, Canyon
	"I really enjoyed it." – Participant 8, Session 5, Canyon
	"It was very good." – Participant 11, Session 5, Canyon
	"It feels good." – Participant 1, Session 4, Countryside
	"I feel so good." – Participant 1, Session 4, Countryside
	"I liked it." – Participant 3, Session 5, Countryside
	"I think it's really quite good." – Participant 8, Session 1, Countryside
	"I like it." – Participant 9, Session 4, Countryside
	"It was really nice." – Participant 9, Session 4, Countryside
	"It's nice, it passes the time." – Participant 9, Session 4, Countryside
	"I like it." – Participant 10, Session 3, Countryside
	"It's a good idea, what you're doing." – Participant 11, Session 3, Countryside
	"It was beautiful and satisfying." – Participant 13, Session 5, Countryside
	"It was pleasant." – Participant 15, Session 2, Countryside
	"It cleared my mind." – Participant 16, Session 5, Countryside
	"It clears your mind, it's great!" – Participant 18, Session 4, Countryside
	"It's original." – Participant 18, Session 2, Countryside
	"It's not bad." – Participant 8, Session 3, Museum
	"It's really good." – Participant 8, Session 3, Museum
	"Thank you, it was super beautiful, I really liked it!" – Participant 8, Session 3, Museum
	"It's really nice!" – Participant 9, Session 2, Museum
	"It was very good." – Participant 17, Session 3, Museum
	"It's good, a nice discovery, it felt real." – Participant 2, Session 4, Oasis
	"I found it very beautiful." – Participant 4, Session 4, Oasis
	"Thank you, it made me feel good, I was able to travel!" – Participant 8, Session 2, Oasis
	"I'm very happy." – Participant 9, Session 1, Oasis
	"Uh, it's nice, really." – Participant 9, Session 1, Oasis
	"It's really beautiful. I'm very happy." – Participant 9, Session 1, Oasis
	"It's nice. Well, thank you! It warms my heart." – Participant 9, Session 1, Oasis
	"It's a change, it entertains me." – Participant 18, Session 3, Oasis
	"Happy." – Participant 19, Session 4, Oasis
	"It's a beautiful montage and a great experience." – Participant 20, Session 1, Oasis
	"It's really beautiful. I really like it, you know." – Participant 5, Session 1, Sea
	"It really makes me happy!" – Participant 5, Session 1, Sea
	"It makes me feel good." – Participant 5, Session 1, Sea
	"I liked it." – Participant 8, Session 4, Sea
	"I like diving, it made me happy." – Participant 8, Session 4, Sea
	"It passes the time." – Participant 9, Session 3, Sea

Subthemes	Verbatims
	“It was good.” – Participant 10, Session 1, Sea
	“It’s very good! I really like all of this.” – Participant 11, Session 2, Sea
	“I really like all of this.” – Participant 11, Session 2, Sea
	“I like this.” – Participant 12, Session 3, Sea
	“It was good, I liked it.” – Participant 15, Session 3, Sea
	“It’s wonderful.” – Participant 16, Session 2, Sea
	“It was good.” – Participant 17, Session 2, Sea
	“Very happy.” – Participant 19, Session 2, Sea
	“I had a great time. It felt good.” – Participant 1, Session 1, Summer Forest
	“I’m happy.” – Participant 10, Session 2, Summer Forest
	“Thanks for the walk.” – Participant 15, Session 5, Summer Forest
	“It was good.” – Participant 17, Session 1, Summer Forest
	“It’s a lovely activity.” – Participant 17, Session 1, Summer Forest
	“Very happy with the moment.” – Participant 19, Session 1, Summer Forest
	“I'm happy that someone is taking care of me.” – Participant 19, Session 1, Summer Forest
	“I appreciated what was offered.” – Participant 1, Session 3, Winter Forest
	“It was very good, I really liked it.” – Participant 9, Session 5, Winter Forest
	“I was at the cinema, it’s really very good!” – Participant 11, Session 4, Winter Forest
	“Happy.” – Participant 19, Session 5, Winter Forest
	“Sad that it’s over.” – Participant 4, Final interview
	“I really liked it! – Participant 5, Final interview
	“It’s interesting and very good.” – Participant 7, Final interview
	“It’s an interesting activity for a nursing home.” – Participant 9, Final interview
	“It helps to clear your mind and escape the everyday.” – Participant 9, Final interview
	“Very happy.” – Participant 10, Final interview
	“It’s beautiful and refreshing.” – Participant 12, Final interview
	“It’s a good activity.” – Participant 13, Final interview
**Sense of calm and relaxation**	“I feel better now.” – Participant 8, Session 5, Canyon
	“An activity like this can calm, brighten, and refresh.” – Participant 20, Session 5, Canyon
	“It was a relaxing activity.” – Participant 4, Session 5, Countryside
	“I feel relaxed, I could spend the afternoon here.” – Participant 5, Session 4, Countryside
	“It’s really relaxing, I like it a lot.” – Participant 8, Session 3, Museum
	“It has a relaxing effect.” – Participant 20, Session 3, Museum
	“It’s relaxing, it makes me feel good.” – Participant 20, Session 3, Museum
	“I close my eyes, it’s restful.” – Participant 3, Session 4, Oasis
	“I feel calm.” – Participant 10, Session 4, Oasis
	“I’m very calm, I even managed to forget your presence.” – Participant 20, Session 1, Oasis
	“It’s very relaxing, I’m very calm.” – Participant 20, Session 1, Oasis
	“I’m very relaxed now.” – Participant 6, Session 4, Sea
	“I feel one thousand percent relaxed!” – Participant 6, Session 4, Sea

Subthemes	Verbatims
	"I feel relaxed despite my pain." – Participant 7, Session 2, Sea
	"I feel relaxed." – Participant 20, Session 4, Sea
	"A bit funny, but relaxed." – Participant 1, Session 1, Summer Forest
	"I'm calm and detached from my worries." – Participant 1, Session 2, Summer Forest
	"Relaxing over time." – Participant 1, Session 5, Summer Forest
	"It relaxes me." – Participant 7, Session 1, Summer Forest
	"I feel relaxed and good." – Participant 20, Session 2, Summer Forest
	"I'm very calm, it's restful and very pretty." – Participant 2, Session 3, Winter Forest
	"I felt good and soothed." – Participant 4, Session 3, Winter Forest
	"It's relaxing. You feel calm in the environment and it's pleasant." – Participant 2, Final interview
	"It was an interesting and relaxing activity." – Participant 4, Final interview
	"It's soothing." – Participant 6, Final interview
	"It's relaxing." – Participant 7, Final interview
	"Soothing and allows you to travel with each session." – Participant 8, Final interview
	"There is a relaxing effect." – Participant 9, Final interview
	"It's an enjoyable and relaxing activity." – Participant 10, Final interview
**Beauty of environments**	"It's beautiful." – Participant 6, Session 5, Canyon
	"It's pretty." – Participant 11, Session 5, Canyon
	"The landscape is impressive." – Participant 2, Session 5, Countryside
	"It's very beautiful." – Participant 3, Session 5, Countryside
	"It's pretty." – Participant 8, Session 1, Countryside
	"It's beautiful." – Participant 8, Session 1, Countryside
	"It was beautiful." – Participant 16, Session 5, Countryside
	"It's splendid, very beautiful, it's a pleasure to see this, it's like a movie, it's beautiful to see the world." – Participant 19, Session 3, Countryside
	"Oh, it's pretty." – Participant 9, Session 2, Museum
	"Wow, it's beautiful." – Participant 9, Session 2, Museum
	"Look how beautiful it is." – Participant 3, Session 4, Oasis
	"It's pretty." – Participant 9, Session 1, Oasis
	"Oh, it's pretty." – Participant 9, Session 1, Oasis
	"It's really pretty." – Participant 9, Session 1, Oasis
	"The landscape was nice." – Participant 15, Session 1, Oasis
	"It's good, it's really well done." – Participant 5, Session 1, Sea
	"It's magnificent." – Participant 19, Session 2, Sea
	"At least here I see beautiful things." – Participant 1, Session 1, Summer Forest
	"It's beautiful, it's pretty." – Participant 7, Session 1, Summer Forest
	"It's really pretty." – Participant 10, Session 2, Summer Forest
	"Very beautiful, unique, I like it." – Participant 19, Session 1, Summer Forest
	"It's well done." – Participant 7, Session 3, Winter Forest
	"It's really nice." – Participant 9, Session 5, Winter Forest
	"It's really pretty." – Participant 9, Session 5, Winter Forest

Subthemes	Verbatims
	"It's beautiful, splendid, unique." – Participant 19, Session 5, Winter Forest<br>"The images are very pretty and realistic." – Participant 7, Final interview
**Appreciation of nature-related visual elements**	"I like seeing the plants at the water's edge." – Participant 16, Session 3, Canyon<br>"It's funny to see those little creatures running around everywhere." – Participant 2, Session 4, Countryside<br>"I really enjoyed the water." – Participant 15, Session 2, Countryside<br>"I like the sand." – Participant 16, Session 1, Oasis<br>"I liked seeing the animals." – Participant 20, Session 1, Oasis<br>"The sea is beautiful." – Participant 3, Session 1, Sea<br>"You can even see the seagulls." – Participant 3, Session 2, Sea<br>"That's beautiful. It makes me happy to see the sea." – Participant 5, Session 1, Sea<br>"The rocks at the water's edge are nice." – Participant 7, Session 2, Sea<br>"Oh, fish, it's beautiful!" – Participant 9, Session 3, Sea<br>"I enjoyed the birds." – Participant 17, Session 2, Sea<br>"The birds are pleasant." – Participant 1, Session 2, Summer Forest<br>"I like the forest, it made me happy." – Participant 11, Session 1, Summer Forest<br>"I really like seeing this nature." – Participant 16, Session 4, Summer Forest<br>"I like the animals." – Participant 19, Session 1, Summer Forest<br>"Oh, the trees, it's beautiful." – Participant 19, Session 1, Summer Forest<br>"I like the changing sun." – Participant 20, Session 2, Summer Forest<br>"Snow has its charm too, nonetheless." – Participant 2, Session 3, Winter Forest<br>"The snowy forest is beautiful like this." – Participant 7, Session 3, Winter Forest<br>"I preferred the sea and oasis environments." – Participant 5, Final interview
**Sense of presence and realism**	"It feels like we're there." – Participant 12, Session 5, Canyon<br>"It feels like we're in Africa." – Participant 15, Session 1, Oasis<br>"It feels like we're in the Sahara." – Participant 19, Session 4, Oasis<br>"It feels like we're in the dunes, by the sea, in Spain." – Participant 19, Session 4, Oasis<br>"It's a very realistic environment." – Participant 20, Session 1, Oasis<br>"It's beautiful, it borders on reality." – Participant 2, Session 2, Sea<br>"It's beautiful, we feel like we're on a boat here." – Participant 7, Session 2, Sea<br>"I appreciated the realism of the images." – Participant 16, Session 2, Sea<br>"You really feel like you're there, it's splendid." – Participant 19, Session 2, Sea<br>"Very realistic." – Participant 1, Session 1, Summer Forest<br>"I felt like I was in the forest." – Participant 1, Session 1, Summer Forest<br>"I walked in the forest." – Participant 12, Session 2, Summer Forest<br>"We felt like we were in the forest." – Participant 12, Session 2, Summer Forest<br>"It's as if I were there." – Participant 13, Session 1, Summer Forest<br>"It was good, we were in the middle of nature." – Participant 15, Session 5, Summer Forest<br>"You really feel like you're there, it's really beautiful." – Participant 13, Session 3, Winter Forest
**Experience of travel and escape**	"It makes you want to be there." – Participant 6, Session 5, Canyon<br>"It was very beautiful, it allowed me to travel." – Participant 2, Session 5, Countryside

Subthemes	Verbatims
	"It's a change of scenery." – Participant 8, Session 1, Countryside
	"I travelled!" – Participant 10, Session 5, Countryside
	"Thank you, it made me feel good, I was able to travel!" – Participant 8, Session 2, Oasis
	"It's really nice, you feel completely transported." – Participant 7, Session 4, Oasis
	"It feels like being in an oasis, in the middle of the desert." – Participant 15, Session 1, Oasis
	"I feel good at the sea." – Participant 3, Session 2, Sea
	"It's nice. I travelled to the sea." – Participant 9, Session 3, Sea
	"I travelled to the sea." – Participant 10, Session 1, Sea
	"As soon as I started moving, I forgot the feeling of being confined, I felt like I was in the forest." – Participant 18, Session 1, Summer Forest
	"It's very much a change of scenery." – Participant 2, Final interview
	"It's great, you travel." – Participant 5, Final interview
	"It allows you to travel." – Participant 6, Final interview
**Wonder and amazement**	"My God, that's amazing." – Participant 8, Session 1, Countryside
	"Wow!" – Participant 9, Session 4, Countryside
	"Oh my." – Participant 9, Session 2, Museum
	"Wow!" – Participant 8, Session 2, Oasis
	"Wow, I wasn't expecting that!" – Participant 9, Session 1, Oasis
	"I wasn't expecting that." – Participant 9, Session 1, Oasis
	"It's still unique." – Participant 19, Session 4, Oasis
	"Wow!" – Participant 9, Session 3, Sea
	"It's impressive, nonetheless." – Participant 1, Session 1, Summer Forest
	"It's unique." – Participant 19, Session 5, Winter Forest
**Admiration for the technology**	"It's impressive." – Participant 3, Session 5, Countryside
	"I wouldn't have wanted to have a headset on my eyes." – Participant 5, Session 4, Countryside
	"Impressed by the setup." – Participant 8, Session 1, Countryside
	"This machine is a great idea." – Participant 4, Session 1, Sea
	"It's impressive what technology allows, nonetheless, it's crazy." – Participant 2, Session 1, Summer Forest
	"Technology is amazing." – Participant 13, Session 1, Summer Forest
	"It's the first time I've seen a set of screens in a room. It's original!" – Participant 19, Session 1, Summer Forest
	"The one who chose the films, is a, great one." – Participant 19, Session 5, Winter Forest
**Appreciation of auditory elements**	"We can sing." – Participant 12, Session 1, Countryside
	"The music is pretty, very soft like that." – Participant 2, Session 4, Oasis
	"I enjoyed the music." – Participant 4, Session 4, Oasis
	"The music sounds like Hollywood." – Participant 7, Session 4, Oasis
	"I hear the sound of the waves." – Participant 3, Session 1, Sea
	"The bird is happy, it sings all the time." – Participant 13, Session 1, Summer Forest
	"It's well done, with the music now." – Participant 7, Session 3, Winter Forest
**Engagement**	
**Curiosity and**	"It's a nice discovery. I didn't know this game." – Participant 18, Session 5, Canyon

Subthemes	Verbatims
**engagement in VR experience**	"Disappointed that the session is over, I want to do it again." – Participant 1, Session 4, Country side
	"It was an interesting activity." – Participant 4, Session 5, Countryside
	"Curious to discover the mountain." – Participant 4, Session 5, Countryside
	"I like everything that is new." – Participant 7, Session 5, Countryside
	"And the depth of the lake, how much is it?" – Participant 19, Session 3, Countryside
	"Fully enjoys the session." – Participant 8, Session 3, Museum
	"It's interesting, it makes me want to discover the next environments." – Participant 20, Session 3, Museum
	"I'm delighted to finally be able to discover the setup." – Participant 6, Session 1, Oasis
	"Where are we going today?" – Participant 7, Session 4, Oasis
	"I'd like to come back, it's interesting." – Participant 8, Session 2, Oasis
	"I'm curious, so I like it." – Participant 15, Session 1, Oasis
	"We can start again whenever you want." – Participant 15, Session 1, Oasis
	"I liked everything, I want to come back." – Participant 3, Session 1, Sea
	"It was good, shall we do it again?" – Participant 19, Session 2, Sea
	"I enjoyed the moment and look forward to coming back when I feel better, in order to enjoy it more." – Participant 1, Session 1, Summer Forest
	"It was good, when do we go back?" – Participant 12, Session 2, Summer Forest
	"I come with pleasure." – Participant 1, Session 3, Winter Forest
	"I had never seen a launch like that." – Participant 19, Session 5, Winter Forest
	"I'm missing it." – Participant 1, Final interview
	"I want to go back, it was very good." – Participant 3, Final interview
	"Disappointed that it's ending." – Participant 5, Final interview
	"It's interesting. We learn things and we travel." – Participant 11, Final interview
**Desire to share the experience with loved ones**	"When I tell my granddaughter, she'll want to come too." – Participant 9, Session 2, Museum
	"I'm going to talk about it with my son and my granddaughter, they're going to be jealous." – Participant 9, Session 1, Oasis
	**Reminiscence**
**Recall of specific personal experi-ences**	"The music we hear, this track, this song, I know its story." – Participant 15, Session 4, Austral ian Road
	"I traveled to this place." – Participant 16, Session 3, Canyon
	"It reminds me of school memories." – Participant 20, Session 3, Museum
	"It made me want to hop on my motorcycle and go for a ride." – Participant 13, Session 4, Oasis
	"I like the forest, because when I was younger, we often went for walks in the forest with my father. And I liked it." – Participant 1, Session 1, Summer Forest
	"I felt rejuvenated, it reminds me of my youth." – Participant 18, Session 1, Summer Forest
	"I've always liked the forest." – Participant 19, Session 1, Summer Forest
	"Is this the Soignies forest here?" – Participant 7, Session 3, Winter Forest
**Recall of memories**	"This reminds me of memories and keeps me occupied." – Participant 9, Session 4, Countryside
	"It reminds me of memories." – Participant 5, Session 1, Sea
	"It reminded me of memories." – Participant 15, Session 3, Sea
	"It reminded me of good memories." – Participant 16, Session 2, Sea

Subthemes	Verbatims
	"It reminds me of beautiful memories." – Participant 19, Session 5, Winter Forest
	"It reminds you of memories." – Participant 5, Final interview
	"It reminds me of memories." – Participant 6, Final interview
	**Negative perceptions**
**Excessive Virtuality and lack of interaction**	"It doesn't feel very real." – Participant 4, Session 5, Countryside
	"I would have liked more interactions." – Participant 6, Session 1, Oasis
	"It's pretty here, but I expected more action." – Participant 6, Session 1, Oasis
	"It's pretty, but very virtual." – Participant 4, Session 2, Summer Forest
	"The images were beautiful, but too virtual." – Participant 4, Session 2, Summer Forest
**Feelings of fear and enclosure**	"The room is too small, I felt trapped." – Participant 12, Session 1, Countryside
	"It's too close, it scares me." – Participant 3, Session 1, Summer Forest
**Decrease in interest**	"Tired." – Participant 1, Session 3, Winter Forest
